# Functionalisation of a heat-derived and bio-inert albumin hydrogel with extracellular matrix by air plasma treatment

**DOI:** 10.1038/s41598-020-69301-7

**Published:** 2020-07-24

**Authors:** John Ong, Junzhe Zhao, Galit Katarivas Levy, James Macdonald, Alexander W. Justin, Athina E. Markaki

**Affiliations:** 1 Department of Engineering, University of Cambridge, Trumpington Street, Cambridge, CB2 1PZ UK; 2 East of England Gastroenterology Speciality Training Program, Fulbourn, Cambridge, CB21 5XB UK; 3 Department of Medicine, National University of Singapore, Kent Ridge Road, Singapore, 119228 Singapore; 4 Duke-NUS Medical School, 8 College Road, Singapore, 169857 Singapore

**Keywords:** Biochemistry, Biological techniques, Biotechnology, Chemistry, Materials science

## Abstract

Albumin-based hydrogels are increasingly attractive in tissue engineering because they provide a xeno-free, biocompatible and potentially patient-specific platform for tissue engineering and drug delivery. The majority of research on albumin hydrogels has focused on bovine serum albumin (BSA), leaving human serum albumin (HSA) comparatively understudied. Different gelation methods are usually employed for HSA and BSA, and variations in the amino acid sequences of HSA and BSA exist; these account for differences in the hydrogel properties. Heat-induced gelation of aqueous HSA is the easiest method of synthesizing HSA hydrogels however hydrogel opacity and poor cell attachment limit their usefulness in downstream applications. Here, a solution to this problem is presented. Stable and translucent HSA hydrogels were created by controlled thermal gelation and the addition of sodium chloride. The resulting bio-inert hydrogel was then subjected to air plasma treatment which functionalised its surface, enabling the attachment of basement membrane matrix (Geltrex). In vitro survival and proliferation studies of foetal human osteoblasts subsequently demonstrated good biocompatibility of functionalised albumin hydrogels compared to untreated samples. Thus, air plasma treatment enables functionalisation of inert heat-derived HSA hydrogels with extracellular matrix proteins and these may be used as a xeno-free platform for biomedical research or cell therapy.

## Introduction

Albumin is an abundant non-glycosylated, 66.4 kDa protein in human serum that has a physiological half-life of approximately 19 days. It is synthesized predominantly by hepatocytes and poorly excreted through the renal glomerulus^1^. Being poorly metabolised and weakly immunogenic, it is stable in vivo. Albumin is also versatile, acting as a weak pH buffer, and as a stabiliser to important proteins, hormones, metal ions, nanoparticles and drugs, making it an attractive biomaterial. These desirable attributes have led to extensive research into albumin as a protein conjugate for drug delivery and pharmacotherapy^1–3^. However, the use of albumin-based hydrogels in biomedical research is comparatively under-studied^4^.

Recently, we demonstrated that human serum albumin (HSA) embedded in a fibrin hydrogel significantly promoted osteoblast differentiation and vascular self-organisation of human endothelial cells^5^. However, high cell numbers (10^6^ cells/ml or greater) degrade these fibrin-based hydrogels within 8–10 days making it unsuitable for studying long term bone development and implantation into animal models. Furthermore, it was anticipated that the implantation of fibrin-based scaffolds into bone would accelerate its degradation in vivo because of the presence of serum fibrinolytics and movement of bone during locomotion. Therefore, albumin-based hydrogels were explored as an alternative material for scaffolds. Heat-derived bovine serum albumin (BSA) hydrogels (20% w/v) exhibit high Young’s modulus (~ 55 kPa) and longer degradation periods (~ 4 to 8 weeks) in vitro and in vivo, however they are completely opaque^6^. Unfortunately there is a paucity of data on heat-derived HSA and approximately 75% of all studies on albumin hydrogels have been conducted on bovine serum albumin (BSA)^4^. This is problematic because BSA and HSA only share 76% similarity in amino acid sequences and this accounts for different properties between the two proteins^7,8^. For example, Arabi et al.^9^ demonstrated that 20% (w/v) HSA and 20% (w/v) BSA solutions have non-identical phase diagrams at acidic pH. Therefore, observations made with BSA hydrogels cannot be directly extrapolated to HSA hydrogels.

Albumin hydrogels are usually derived by three main methods: heat induced gelation, pH induced gelation and chemical crosslinking. Each method affects the properties of the resulting hydrogels, e.g. gelation precipitated by strongly acidic or strongly alkali pH produces transparent albumin hydrogels whereas heat induced gelation produces an opaque hydrogel with larger pore sizes and a longer biodegradation time^6,10^. The most commonly used method is chemical crosslinking, often with glutaraldehyde or conjugated with functionalised polyethylene glycol (PEG)^4^. However these processes are laborious, costly and the resulting scaffolds can be immunogenic^4,11^. Heat-induced gelation is by far the easiest method to synthesize albumin hydrogels, however the opacity of resulting hydrogels precludes normal microscopy often required for cell and tissue culture. In addition, conformational differences at various pHs and temperatures restrict protein binding sites contributing to poor cell attachment onto hydrogel surfaces.

In order for heat-derived HSA hydrogels to be useful for biological applications, novel methods to improve better hydrogel clarity and cell attachment are required. To that end, we successfully adapted a method^12^ previously used to obtain transparent BSA hydrogels with the addition of sodium chloride (NaCl) to produce translucent albumin hydrogels. However despite achieving sufficient hydrogel clarity, cell attachment to heat-derived albumin hydrogel surfaces remained poor. Therefore, functionalisation of hydrogel surfaces by air plasma treatment was explored because of the limited flexibility within the thermal gelation method. During air plasma treatment, atmospheric gases such as oxygen, hydrogen or nitrogen, are ionised with a strong radio-frequency (RF) electromagnetic field to form a non-thermal plasma. Electrons are excited to very high temperatures (~ 10^5^–10^6^ K) while collisions with the background ions maintain the plasma at near ambient temperatures overall^13^. These charged species introduce reactive groups onto the surfaces of polymeric materials such as carbonyl, carboxyl and hydroxyl functionalities. Consequently, these functional groups can bind extracellular matrices or cell surface proteins onto the plasma-treated surface.

While plasma-activated bonding has become a routine procedure in the assembly of microfluidic devices and microelectromechanical systems (MEMS), the treatment of tissue engineering scaffolds with plasma is still gaining attention, particularly in bone tissue engineering^14–20^. Biomaterials such as silk, gelatin, chitosan, hydroxyapatite, calcium phosphate and polycaprolactone have been successfully plasma treated, however, to the best of our knowledge, plasma treatment of albumin hydrogels has not been previously reported. Importantly, unlike these materials HSA offers added advantages of being a xeno-free polymer that can be patient derived for easier transition into downstream clinical applications such as platforms for cell therapy, drug delivery or scaffolds for tissue engineering.

Therefore, the aims of this work were to (1) create a translucent heat-derived HSA hydrogel and (2) improve cell attachment to heat-derived HSA hydrogels by functionalising them with bioactive, extracellular matrices. Below, novel methods for obtaining translucent HSA hydrogels and their functionalisation by air plasma treatment are described. The successful coating of heat-derived HSA hydrogels with basement membrane matrix (Geltrex) and the stable culture of foetal human osteoblasts (fHObs) in vitro are then demonstrated on plasma treated, matrix-coated hydrogels and not on untreated, matrix-coated hydrogels. The results herein provide proof of concept that heat-derived HSA hydrogels can be functionalised inexpensively by air plasma treatment, providing a xeno-free biocompatible material for biomedical research, especially for tissue engineering and emerging cell therapies.

## Results

### Type of solvent, HSA concentration, heating temperatures and duration affected hydrogel opacity

**Figure 1:**
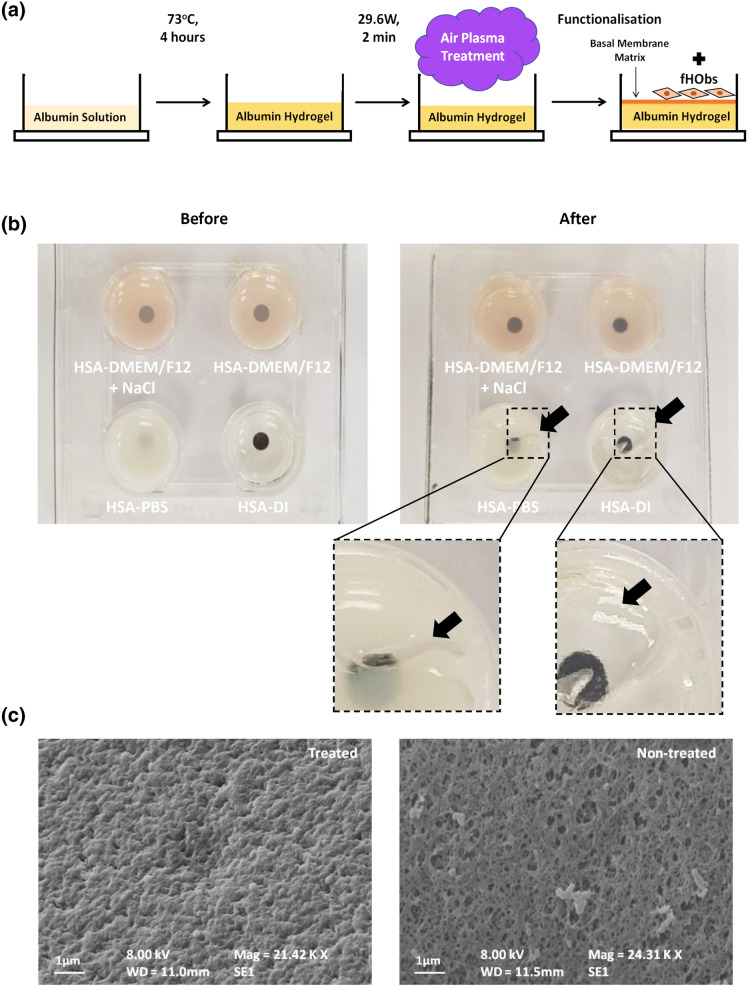
(**a**) Schematic representation of hydrogel preparation and functionalisation. (**b**) Hydrogels formed by dissolving HSA in DMEM/F12 with additional sodium chloride (top left), DMEM/F12 (top right), PBS (bottom left) and DI (bottom right). Left panel: before plasma treatment, right panel: after plasma treatment. Black dots printed on paper beneath the well-plate to demonstrate hydrogel opacity. Black arrows point at regions where the hydrogels failed after plasma treatment. (**c**) SEM images of HSA-DMEM/F12 hydrogel surfaces; plasma-treated and non-plasma treated surfaces.

HSA was dissolved in solutions with different ionic content: deionised water (DI), Dulbecco's Phosphate Buffered Saline (PBS) and Dulbecco's Modified Eagle Medium nutrient mixture F-12 (DMEM/F12), at a pH range suitable for normal cell culture (pH 7.1─7.4). Controlled heat was then applied to the solutions followed by air plasma treatment and surface coating as illustrated in Fig. 1a. Briefly, hydrogel opacity increased with increasing albumin concentration and 10% w/v HSA was found to be optimal for hydrogel clarity (Supplementary Table S1). Screening experiments further demonstrated that heating aqueous solutions of 10% w/v albumin above 75 °C for 60 min produced clear to translucent HSA-DI hydrogels, but opaque HSA-PBS and HSA-DMEM/F12 hydrogels, rendering the latter two hydrogels unsuitable for bright-field microscopy and cell culture. Heating at 70–75 °C for 60 min resulted in clear HSA-DI hydrogels, translucent HSA-DMEM/F12 hydrogels and opaque HSA-PBS hydrogels (Fig. 1b, left panel). Below 70 °C, all three solutions failed to gel within 60 min. An array of gelation conditions was determined (Supplementary Table S2) and the optimal heating temperature and duration that produced mechanically stable hydrogel were found to be 70–75 °C and four hours respectively.

### Addition of sodium chloride improved the resilience of HSA-DMEM/F12 hydrogels

Although HSA-DI and HSA-PBS hydrogels were obtained under optimal conditions, these were prone to damage during and after plasma treatment (Fig. 1b, right panel). HSA-DMEM/F12 hydrogels, although weak, were resistant to fracturing during plasma treatment, however these were still damaged within 1–3 days of cell culture. Previously, Murata et al.^12^ demonstrated that the addition of sodium chloride to BSA solutions could reduce the opacity of heat-derived BSA hydrogels and facilitate gelation. In this study, it was observed that the addition of 30 mM NaCl to HSA-DMEM/F12, raising the final NaCl concentration to 150 mM, did not result in a noticeable improvement to hydrogel transparency, however it increased hydrogel strength which allowed mechanical testing to be performed subsequently. A significant reduction in fractures during plasma treatment and cell culture were also noted in HSA-DMEM/F12 hydrogels enriched with NaCl. Optical clarity of HSA-PBS hydrogels and the stability of HSA-DI hydrogels were not improved by the addition of NaCl to both solvents (Supplementary Table S3).

### Plasma treatment affects surface roughness of HSA-DMEM/F12 hydrogels

**Figure 2:**
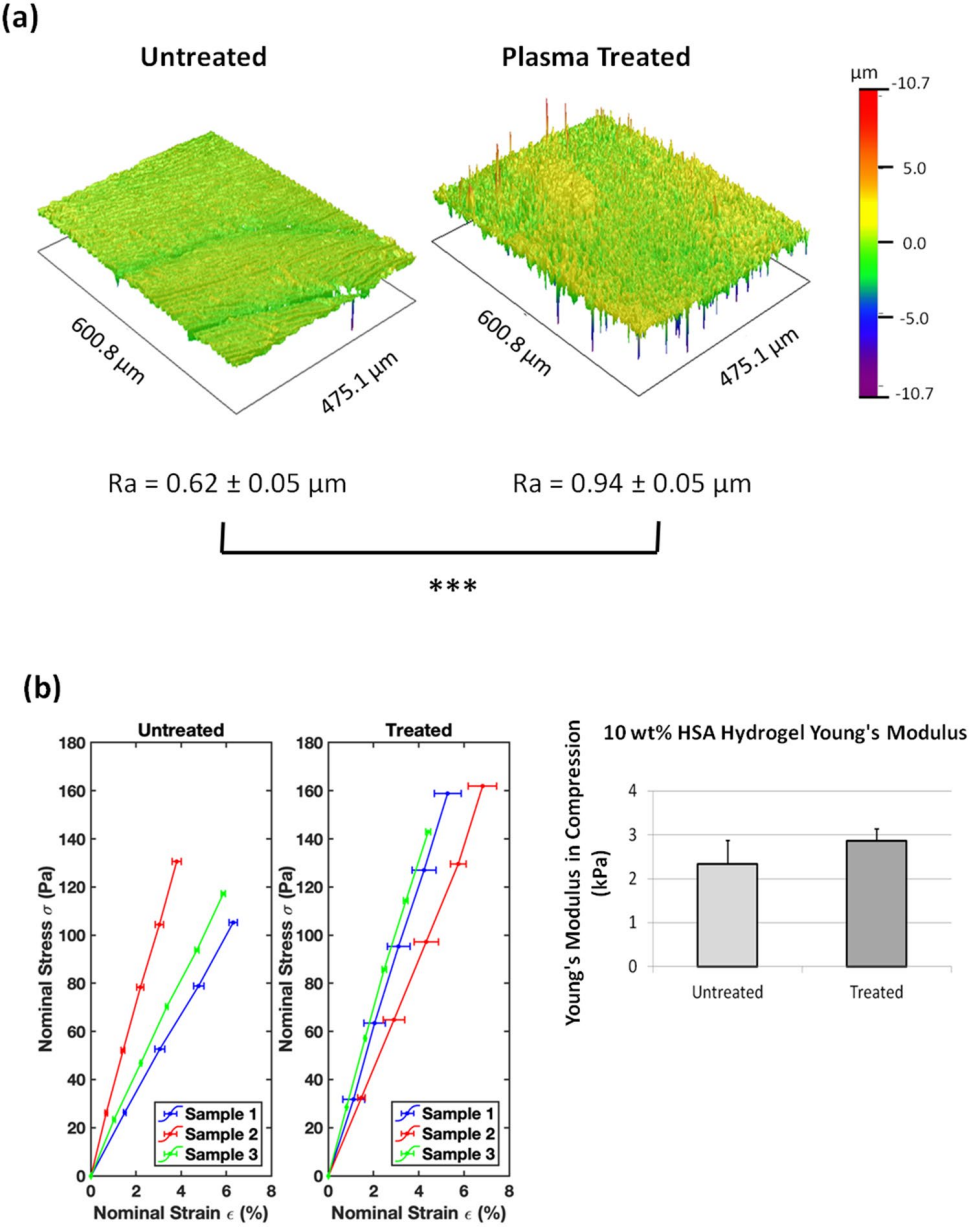
(**a**) Reconstructed surface images of plasma-treated and untreated HSA-DMEM/F12 hydrogels with interferometry. Plasma-treated surfaces are significantly rougher than untreated surfaces, Ra values expressed as mean ± standard deviation, ***p < 0.001. (**b**) Stress–strain curves of plasma-treated and untreated HSA-DMEM/F12 hydrogels with the calculated Young's moduli. Values represent mean ± standard error. No statistical difference was detected between both groups.

Images obtained by scanning electron microscopy (SEM) demonstrated that plasma treatment increased the surface roughness of heat derived HSA-DMEM/F12 hydrogels whereas untreated surfaces had a smoother but porous surface (Fig. 1c). Profile roughness parameters (Ra) obtained by interferometry also confirmed a significant difference between plasma treated and non-plasma treated HSA-DMEM/F12 hydrogel surfaces as shown in Fig. 2a. Nonetheless, both hydrogels were observed to have hydrophilic surfaces with sessile water droplet contact angles below 90°. Plasma treated surfaces, however, were observed to have smaller contact angles than untreated surfaces, suggesting the former is more hydrophilic; 45.7° ± 5.3° vs. 66.8° ± 1.6° respectively, *p* < 0.001 (Supplementary Table S4). Young’s modulus measurements show that untreated and plasma treated HSA-DMEM hydrogels have similar Young's moduli; 2.34 ± 0.54 kPa vs. 2.87 ± 0.27 kPa respectively, *p* = 0.51 (Fig. 2b).

### Plasma treatment enables the coating of HSA-DMEM/F12 hydrogel with basement membrane proteins

**Figure 3:**
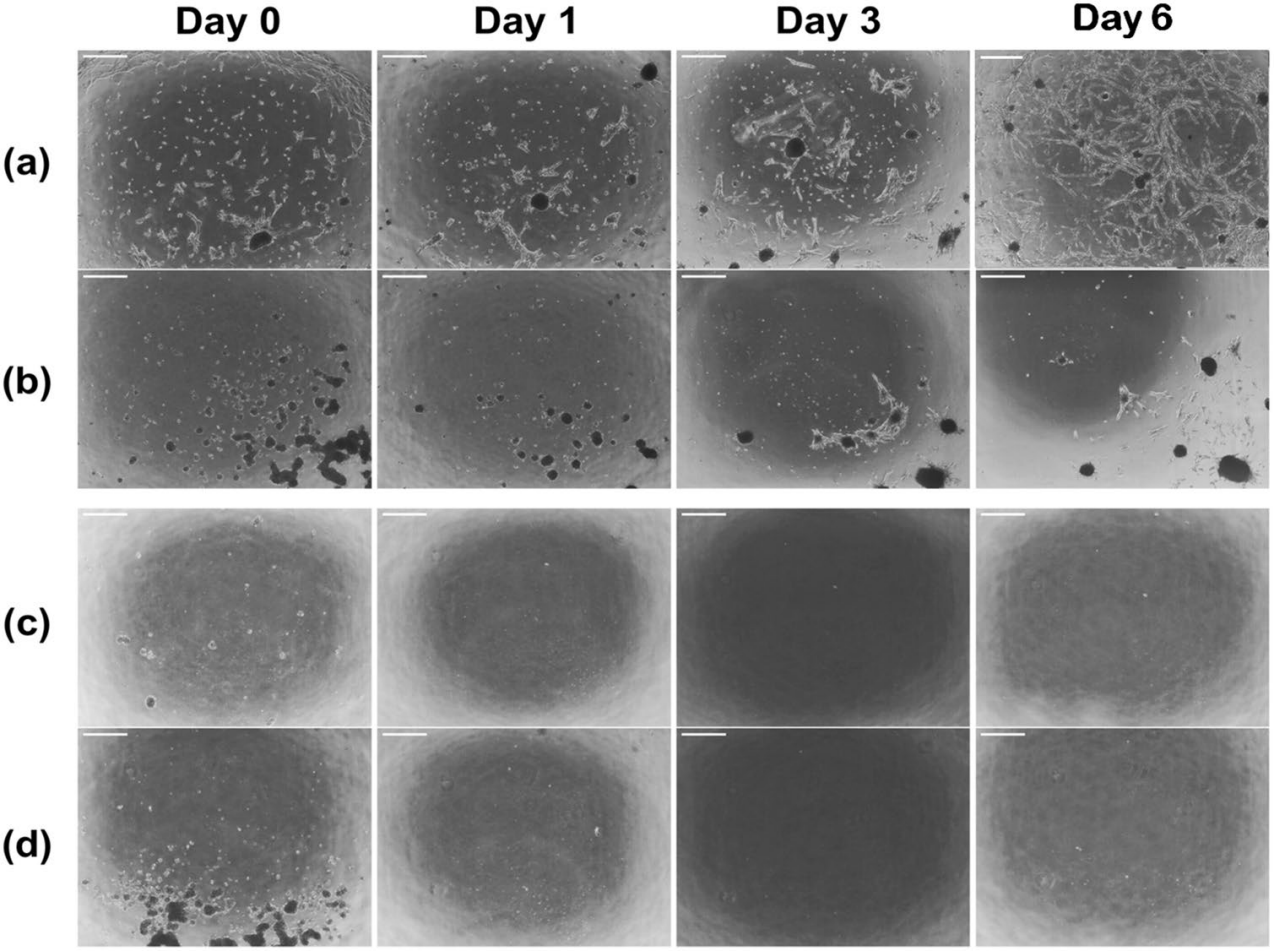
Serial bright field microscopy of fHObs seeded on HSA-DMEM/F12 hydrogels: (**a**) plasma-treated and Geltrex coated surface, (**b**) plasma-treated and uncoated surface, (**c**) non-plasma treated but Geltrex coated surface and (**d**) non-plasma treated and uncoated surface. No cells adhered to (**c**) and (**d**) after 48 h. Cell attachment in (**b**) was poor throughout. Scale bar: 100 µm.

Plasma-treated HSA-DMEM/F12 hydrogels coated with basement membrane proteins (Geltrex), facilitated good attachment of fHObs and supported cell growth (Fig. 3a). Without a surface coating of Geltrex, cell attachment of fHObs on plasma treated HSA-DMEM hydrogel was poor (Fig. 3b). After 6 days of cell culture, cell numbers compared to the initial seeding density on plasma-treated Geltrex-coated HSA-DMEM hydrogels had a mean fold increase of 6.3 ± 0.5. However, cell numbers on plasma-treated uncoated HSA-DMEM hydrogels were observed to be less than the original seeding density: mean fold increase = 0.3 ± 0.1. Differences were statistically significant (p < 0.001). Despite extended cell culture of 8 days, cell confluence remained poor in plasma-treated, uncoated surfaces and fHObs continued to detach with every routine medium change. On non-plasma treated HSA-DMEM/F12 hydrogels, attachment of fHObs to Geltrex coated (Fig. 3c) and uncoated (Fig. 3d) hydrogel surfaces were extremely poor. 48 h after plating fHObs onto these surfaces, no cells remained. These results demonstrate that surface coating of Geltrex onto plasma treated DMEM-HSA hydrogel surfaces was adequately achieved, which allowed the attachment and proliferation of fHObs.

### fHObs cultured on plasma-treated Geltrex-coated HSA hydrogels maintained common osteoblastic signatures

**Figure 4:**
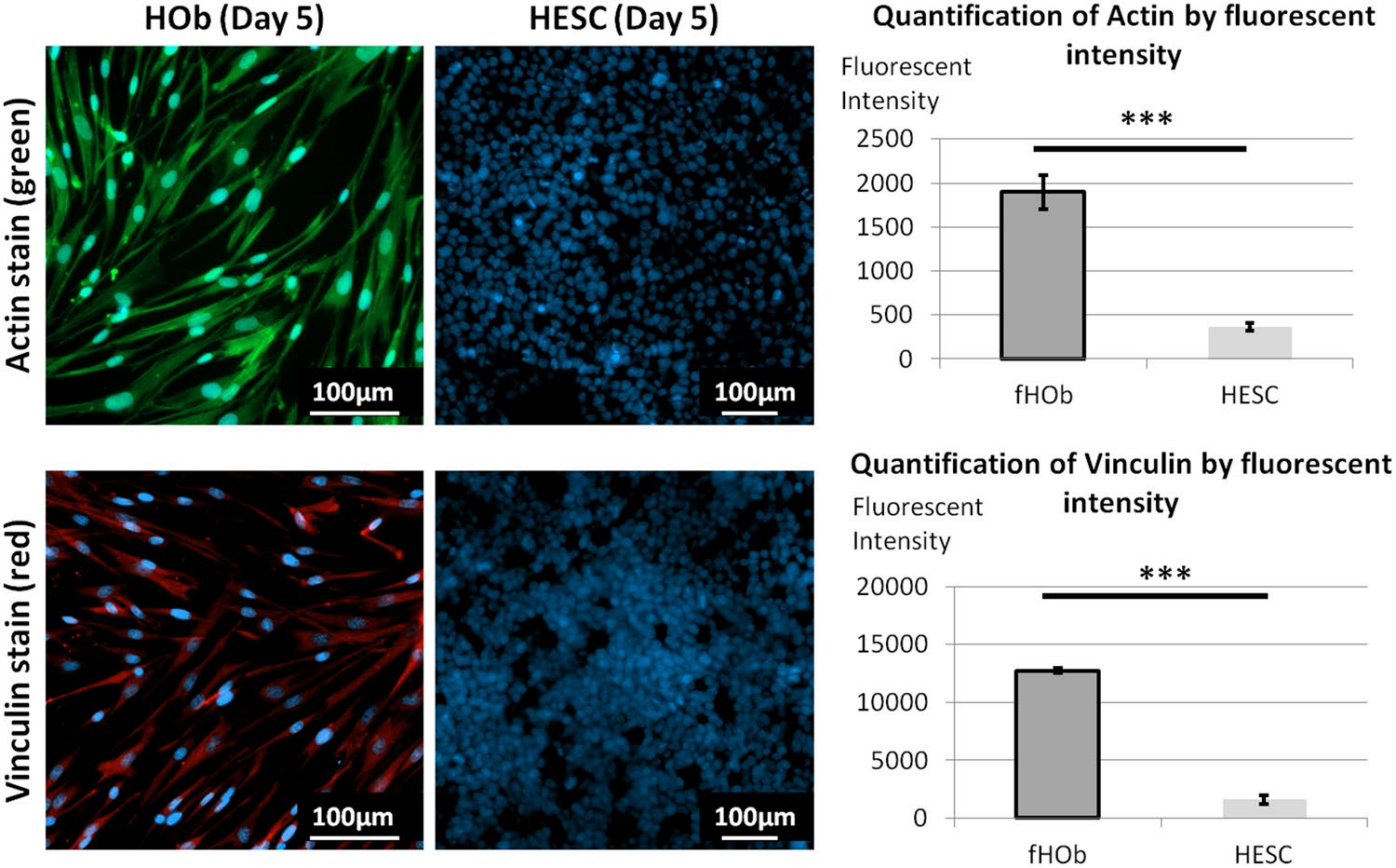
Immunofluorescence imaging and image analysis of fHObs and hESCs cultured on plasma-treated and Geltrex-coated HSA-DMEM/F12 hydrogels. Cytoplasmic staining of actin (green) and vinculin (red). Nuclear staining with DAPI (blue). Statistically significant differences were displayed as ***p < 0.001, and derived by independent t-tests. Data represented as mean ± standard deviation from three independent experiments.

**Figure 5:**
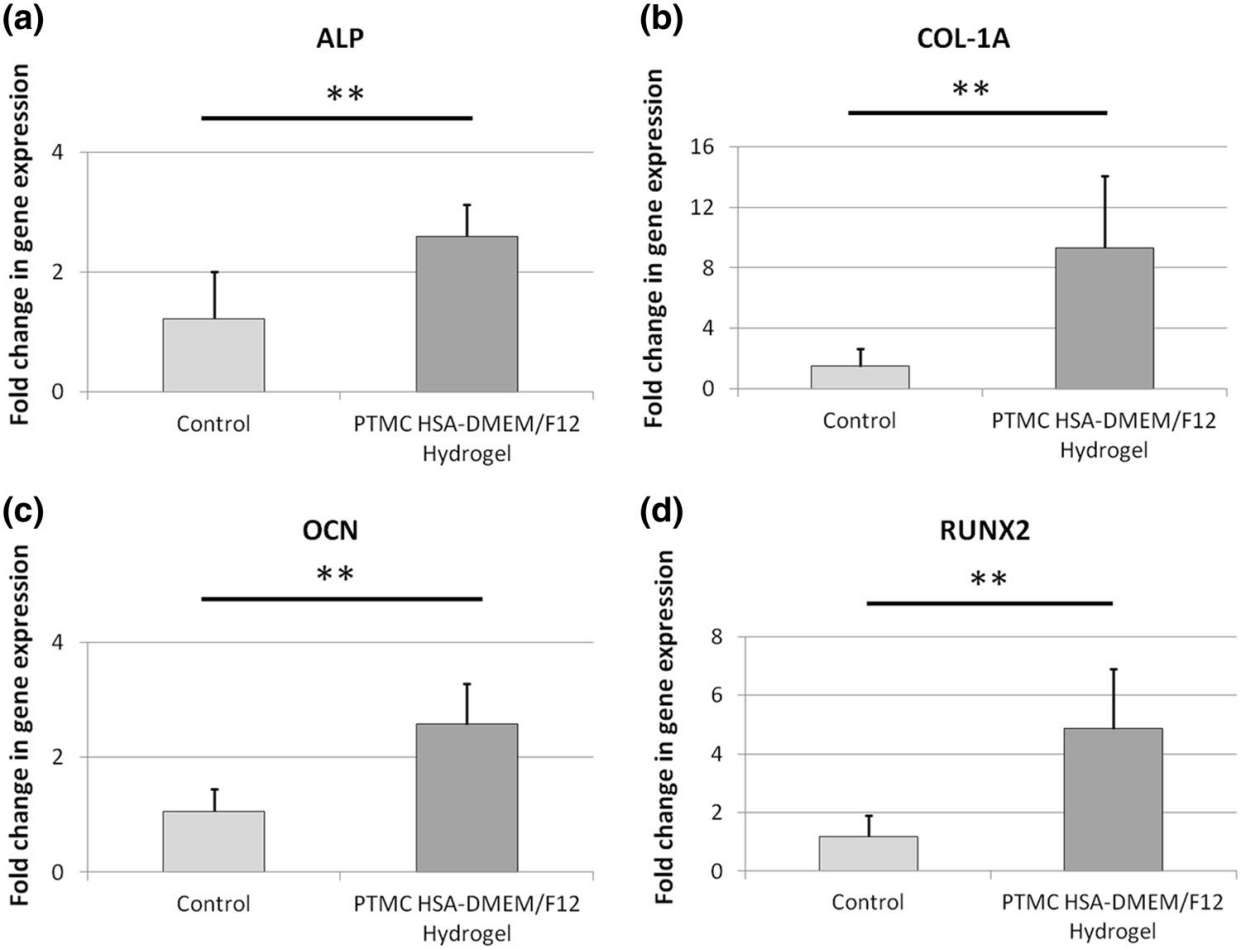
Gene expression analysis of fHObs cultured on plasma-treated, matrix-coated (PTMC) HSA-DMEM/F12 hydrogels at day 8 of culture (**a**) ALP, (**b**) COL1-A1, (**c**) OCN and (**d**) RUNX2. Control samples are fHObs cultured on tissue cultured treated plates and harvested at Day 1. Due to poor cell attachment, insufficient RNA was extracted from fHObs cultured on plasma-treated, uncoated HSA-DMEM/F12 hydrogels. Statistically significant differences were displayed as **p < 0.01, and derived by independent t-tests. Data represented as mean ± standard deviation.

High expression of actin is observed in human osteoblasts^21^ and low actin expression is observed in undifferentiated human embryonic stem cells (hESCs)^22^. Similarly, high vinculin expression is indicative of good osteoblasts adhesion^23^ and vinculin is poorly expressed in stem cells on compliant substrates (< 200 kPa)^24,25^. Comparisons of fluorescent intensities between fHObs and hESCs cultured on plasma-treated, Geltrex-coated HSA-DMEM/F12 hydrogels demonstrated strongly positive actin and vinculin staining in fHObs and conversely poor staining in hESCs (Fig. 4). Further analysis of fHObs cultured on plasma-treated Geltrex-coated HSA hydrogels at Day 8 for alkaline phosphatase (ALP), collagen-1 alpha 1 (COL1A1), osteocalcin (OCN) and runt-related transcription factor 2 (RUNX2), demonstrated preserved and higher gene expression profiles of these commonly expressed osteoblast genes when compared to fHObs cultured on tissue culture treated surfaces at Day 1 (Fig. 5).

## Discussion

The resulting Young's modulus of translucent HSA-DMEM/F12 hydrogels is significantly lower than opaque BSA-PBS hydrogels previously reported; ~ 3 kPa vs. ~ 55kPa^6^ respectively. This is most likely caused by differences in the albumin protein and the concentration that was used for hydrogel synthesis. Although HSA hydrogels were initially investigated for durability, this study prioritised optical clarity over the stiffness of HSA hydrogels to increase its usefulness in biomedical research. A Young's modulus of 3 kPa is identical to the stiffness of many organs in the human body but it is significantly less than bone (trabecular bone ~  ≥ 10GkPa). Nonetheless, these scaffolds remain applicable to research in bone and examples of their potential uses are discussed below. Importantly, the methods described herein above provide a novel, uncomplicated and inexpensive alternative to chemically crosslinking albumin hydrogels which can cause various degrees of local inflammation and fibrous capsule formation after implantation^26,27^.

Ionic content affects hydrogel properties and we observed that increasing the ionic content of HSA-DMEM/F12 by the addition of NaCl significantly improved the rate of gelation and hydrogel strength. In addition, a positive effect was noted when NaCl was added to DI, however this was to a much lesser extent; resulting HSA-DI hydrogels were still prone to damage after handling and air plasma treatment (Supplementary Table S3). Unfortunately, increasing NaCl concentrations resulted in increasing opacity across all hydrogels types. The mechanism of gelation for heat-derived albumin hydrogels are well described in literature^28–31^, and the differences observed between these three hydrogels are most likely explained by the differences in ionic content and concentrations in all three solvents. However, it was not feasible to determine the effect of specific ions on gelation and hydrogel clarity because of the sheer multitude of permutations between ion combinations and concentrations. For example, DMEM/F12 contains up to sixty eight different constituents at varying concentrations. Therefore, only the optimal conditions were described herein.

Our results demonstrated that air plasma treatment alters the surface properties of heat-derived albumin hydrogels allowing functionalisation with target proteins. Importantly, we observed that the degree of air plasma treatment, both intensity and duration of treatment, significantly influenced the degree of functionalisation and hydrogel appearance (Supplementary Table S5). 'Over-treatment' caused macroporous hydrogels which obliterated cell visibility on bright-field microscopy, and 'under-treatment' led to poor functionalisation and cell attachment. It was also observed that the bulk of the hydrogels were influenced by the plasma treatment conditions. While HSA-DI hydrogels were readily damaged during handling even before the plasma treatment, both HSA-PBS hydrogels and HSA-DMEM/F12 hydrogels without NaCl became more prone to damage after the treatment. The difference in ionic content is an important factor in accounting for these differences but the formation of cracks may also be attributed to the fact that air plasma treatment involves rapid changes in temperature, pressure, and potential surface damage from energetic plasma ions. Nonetheless, a limitation of this method is that functionalisation occurs superficially. As a result, the bulk of the hydrogel may remain inert and conjugation of proteins within the albumin hydrogel may not occur. To overcome this limitation, functionalised 3D constructs can be created from assembled planar components. For example, thin albumin hydrogel sheets with a patterned surface could be created through sacrificial moulding, plasma treated, coated with selected matrix proteins, then adhered together with human fibrin to create a 3D construct with organised interconnecting spaces. Such scaffolds could then be investigated for further use in bone disease such as non-union of long bone fractures or in reducing aseptic loosening in bone prostheses.

Similarly, 3D porous constructs have been used in liver tissue engineering to optimise cell delivery for bridging therapy in liver failure, disease modelling and drug studies. These are typically made from PEG and coated with extracellular matrix proteins. Although they have been shown to improve hepatocyte function^32–34^, the biodegradability and acquired immunogenicity of PEG scaffolds^11^ in vivo remain outstanding issues. The observed Young's modulus of HSA-DMEM/F12 hydrogels (2.87 ± 0.27 kPa) was found to be similar to the normal stiffness of human liver tissue which is approximately 2–6 kPa^35–37^. Therefore, HSA-DMEM/F12 hydrogels may provide an alternative to synthetic or de-cellularised liver scaffolds since they are xeno-free, patient-derivable and mimic normal liver stiffness. Apart from the liver, HSA-DMEM/F12 scaffolds may also be used for drug studies and modelling tissue growth for the human heart. Although native heart tissue has higher stiffness than HSA-DMEM/F12 hydrogels, several studies have reported that matrix-coated albumin hydrogels (derived by more laborious methods of electro-spinning and pH induced gelation) have a positive effect on neonatal ventricular rat cardiomyocytes and human cardiomyocytes derived from induced pluripotent stem cells when compared to conventional 2D culture methods^38–41^.

Cell behaviour and function are critically affected by extracellular cues known as the 'niche' which includes extracellular matrix proteins. For bone, laminins and collagen play an integral part of the bone marrow niche which influences bone development and regeneration. For this reason, Geltrex was used as a coating matrix in this study since laminins and collagen are components of Geltrex. Laminins, through various integrin binding sites, suppress osteoclastogenic activity^42^, promote bone marrow mesenchymal stromal cell adhesion and growth^43^, and enhance osteogenic differentiation^44^. Likewise, collagen has also been reported to enhance osteogenic differentiation^44^. An important observation of this study is that plasma treated, matrix-coated albumin hydrogels supported the in vitro attachment and expansion of fHObs similarly. Immunostaining demonstrated expectedly high expression of actin and vinculin in fHObs when compared to HESCs. HESCs were used as a comparison because expression of these proteins are low in undifferentiated stem cells, and also because other cell types such as fibroblasts can express high levels of actin and vinculin.

In vitro studies have demonstrated that osteoblasts exhibit a gradient of maturity and cell behaviour^45,46^. These are affected by factors including, but not limited to, the region of bone and donor age^47^. The presence of actin and vinculin, in addition to the higher gene expression of COL-1A1, ALP, OCN and RUNX2 in fHObs cultured on plasma-treated, Geltrex-coated albumin hydrogels, suggest that fHObs adhered to the surface-coated matrix and this resulted in the observed effect. These early data suggest that Geltrex may enhance the differentiation of immature fHObs towards a more mature phenotype. In particular, RUNX2 is a transcription factor that is critical for osteoblast proliferation and differentiation, especially during the exit of preosteoblasts from the cell cycle and during the maturation of osteoblasts^48,49^. In the latter, RUNX2 expression increases as cells differentiate from osteoprogenitors, to immature osteoblasts, and remains up-regulated to at least when immature osteoblasts become early mature osteoblasts^50^ as general consensus allows. Beyond this time point, views on the expression of RUNX2 and its role in mature osteoblasts are controversial^45,46,49^.

Interestingly a small numbers of fHObs were observed to be able to attach to plasma treated, uncoated HSA-DMEM/F12 hydrogels but these were not able to proliferate. This may be explained by the weak non-specific binding of cell surface receptors or proteins to the functional groups on HSA-DMEM/F12 hydrogels generated by plasma treatment. Conversely, when basement membrane proteins are added to these plasma-treated surfaces, they may bind to the functional groups on the basal surface, and present protein binding domains on the apical surface to facilitate better cell attachment and cellular responses e.g. through integrin binding sites and its downstream pathways.

Lastly, this study had several limitations. All biomarkers used in this study are not specific to bone tissue since elevated levels can be found in, but not limited to, brain and gastrointestinal tissue (https://www.proteinatlas.org). Specific biomarkers for human bone (and osteoblasts) do not exist and the expression profile of these genes and proteins are widely accepted as important characteristics of osteoblast phenotypes. Although Geltrex is commonly used for the culture of human cells, it is derived from murine cancer matrix. Therefore, it may not have any significant physiological relevance to bone development in humans. Laminins and collagens, which are found in abundance in human bone marrrow, are also constituents of Geltrex. As the primary objective of this study was to demonstrate the functionalisation of inert albumin hydrogels with extracellular matrix proteins using air plasma treatment, the use of Geltrex in this context was considered appropriate. In future experiments, now that proof-of-concept has been established, the authors will create 3D xeno-free constructs using recombinant human laminins, collagen, HSA hydrogels and co-cultures to determine the likeness of matrix-enhanced osteoblasts compared with patient-derived adult primary osteoblasts using in vitro and in vivo functional assays.

## Conclusions

In summary, this study has shown that it is possible to obtain translucent and mechanically stable heat-derived HSA hydrogels which can be functionalised with protein matrices through air plasma treatment. These scaffolds can readily support cell culture for biomedical research. Future research is required to study the effect of xeno-free albumin constructs in humanised animal models and determine its suitability in clinical applications such as cell therapy.

## Materials and methods

### Hydrogel synthesis

Human albumin (Sigma A1653) was dissolved in either deionized water, DBPS (Sigma, D8537), or DMEM/F12 (Fisher Scientific, 11574546). DMEM/F12 was supplemented with 1% Penicillin/Streptomycin (Sigma TMS-AB2-C). Human albumin dissolved in DMEM/F12 pre-warmed to 37 °C to make up a 10% (w/v) human albumin solution was used for in vitro experiments. Once filtered, a small volume from a sterile stock solution of 5 M NaCl (Sigma, S5886) was added to the 10% HSA to raise the molarity of NaCl by 30 mM, giving a final approximate NaCl molarity of 150 mM. The solution was then syringe filtered with a 0.1 µm syringe filter (Millipore SLVV033RS) for sterility.

Depending on the experiments, the final solution was added to either 96 well plates (Falcon 351172, 50 µl solution added) or 4 well plates (Thermoscientific 179830, 500 µl solution added) and sealed with aluminium plate sealers (Greiner Bio-One, 676090). Samples were then transferred into an oven and the temperature was raised to 73 °C. Samples were left for 4 h to gel and at the end of the heating process, translucent HSA hydrogels were obtained.

### Air plasma treatment and functionalisation

Hydrogels were transferred *immediately* into a plasma cleaner (Harrick Plasma, PDC-002) after 4 h of heating. Without letting the hydrogel cool, vacuum suction was applied to the chamber for 3 min and high intensity (29.6 W) plasma treatment was then initiated and applied for 2 min under a low inflow of air. Thereafter, well plates containing the treated hydrogels were sealed until ready for use. Note: Plasma treatment causes a rapid reduction in the temperature of the hydrogel, which through ice crystal formation, yields a macroporous gel and makes the visualisation of cells difficult. This is further exacerbated by extended plasma treatment times.

For functionalisation of HSA hydrogels with basement membrane proteins, Geltrex (Life Technologies, A1413302) was used. HSA hydrogels prepared above were first washed with PBS; 500 µl per well for a 4-well plate and 100 µl per well for a 96 well plate. After the aspiration of PBS, Geltrex diluted in DMEM/F12 to a concentration of 2 mg/ml was then added at a volume of 500 µl per well for a 4-well plate and 50 µl per well for a 96 well plate. Samples were then left in the incubator at 37 °C for two hours for surface coating.

### Measurement of Young's modulus

The Young’s modulus (*E*) of the albumin hydrogels was measured in compression using a modified ‘see-saw’ method^51^. The two arms of the see-saw were first balanced before testing. Counterweights were then added onto the free-extending arm to match the height of the loading platen with the sample height. The load was ramped up at a constant rate of 10^–3^ N s^−1^, by pumping liquid via a syringe pump (PHD ULTRA Harvard Apparatus) into a container fixed on top of the loading platen. The resulting displacement was monitored using a biaxial laser micrometer (Keyence, TM-040). Data analysis was conducted in MATLAB (MathWorks). Nominal stresses (*σ*) and strains (*ε*) were plotted, and a line was least square fitted to the data points, which gave an apparent *E* in compression. 4 samples were tested for each group to calculate the mean and standard error of *E*.

### Cell culture

A human fetal osteoblast cell line obtained from the Cell Applications Inc. (406K-05f) was used. Cells were maintained in McCoy's 5A medium (Gibco, 16600082) which was supplemented with heat-inactivated 10% FBS (Invitrogen, 10108-157), 1% Antibiotic-Antimycotic (Gibco, 15240062), 50 mg/ml L-Ascorbic Acid Phosphate and Magnesium Salt (Fujifilm Wako Chemical corporation, 013-19641). As a comparison for immuno-staining, clinical grade human embryonic stem cell (hESC) line KCL-033, a kind gift from Dr Tamir Rashid (King's College London), was used. hESCs were deemed a good comparison because of its low actin and vinculin expression on compliant substrates^22,24,25^. hESCs were cultured on Geltrex and maintained in mTESR Plus media (Stemcell Technologies, 05825) supplemented with 1% penicillin and streptomycin (Sigma, TMS-AB2-C). Accutase (Sigma, A6964) was used to detach both the fHObs and hESCs for passaging and seeding. Passage 3–5 fHObs and passage 25–30 hESCs were used for all experiments. Seeding densities were 40,000 cells per well in a 96 well plate or 400,000 cells per well in a 4 well plate.

### Scanning electron microscopy (SEM)

Plasma treated and non-treated HSA hydrogel samples were prepared for SEM by first fixing in 10% formalin (VWR International, 11699455) under a fume hood overnight. Samples were then thrice washed with PBS before being dehydrated in increasing concentrations of ethanol:DI mixtures; 30, 50, 70, 80, 90, 95 and 100% ethanol. The last step involving 100% ethanol was repeated twice. Samples were submerged in each ethanol:DI mixture for 30 min. For drying, dehydrated samples were submerged in increasing concentrations of hexamethyldisilazane (HMDS):ethanol mixtures under a fume hood; 33.3, 66.6 and 100% HMDS respectively. Samples were submerged in each HMDS:ethanol mixture for 40 min and the last step involving 100% HMDS was repeated twice. Samples were left submerged in 100% HMDS overnight in a fume hood until completely dry the following day. After drying, samples were gold plated prior to imaging. The surface morphology of the samples was observed using a Zeiss Evo LS 15 scanning electron microscope.

### Surface roughness measurements

Surface roughness was measured using a Veeco Wyko NT3300 optical profiler and interferometer. Multiple surface roughness measurements were made on plasma treated and non-plasma treated HSA-DMEM/F12 hydrogels using a × 10 objective. Reconstructed topographical images of the hydrogel surfaces were subsequently obtained.

### Immunofluorescent staining

Firstly, biological samples were fixed with 4% (w/v) paraformaldehyde (Sigma, 252549) for 20 min at 4 °C then washed twice with PBS. A permeabilizing and blocking solution comprising 0.1% Triton X-100 (Sigma, T878), 0.1% TWEEN-20 (Sigma, 125 P6585) and 1% w/v BSA (Sigma, A2153) dissolved in PBS was then added to the samples and left for 30 min at room temperature. 3% donkey serum was added to the blocking solution for unconjugated antibodies (anti-vinculin).

Conjugated FITC-labelled phalloidin (Sigma, P5282) at a dilution of 1:100 was added and left at room temperature for 2 h to visualise cytoplasmic actin. Samples were then washed twice with PBS and NucBlue (Invitrogen R37606) was added to visualise cell nuclei. Samples were left for 30 min before images were acquired. To visualise cytoplasmic vinculin, mouse anti-human vinculin primary antibody (Sigma, V9131) was diluted to 1:100 and added to blocked samples for 2 h at room temperature. Subsequently, two washes with PBS were done and donkey anti-mouse Alexa Fluor 647 secondary antibody (Sigma, A32787) diluted to 1:1,000 was added. Samples were left for another 2 h at room temperature then washed twice with PBS. NucBlue (Invitrogen R37606) was added and samples were left for 30 min before images were acquired.

### Image acquisition and image analysis

After the staining process was complete, images were acquired by the high content screening system Opera Phenix (Perkin Elmer). Image acquisition settings were fixed at 95% excitation and 200 ms exposure. Harmony high-content analysis (HCA) software version 4.9 (Perkin Elmer, HH17000010) was used to measure the fluorescent intensities of cytoplasmic actin and vinculin, and the mean fluorescent intensity was calculated.

### Cell proliferation analysis

Fold increase was determined by the total number of cells per well at Day 6 (C_6_) divided by the total number of cells seeded per well at Day 0 (C_0_): C_6_ ÷ C_0_. Cells were counted manually on Day 0 before seeding. Cells were counted by imaging (Harmony HCA software version 4.9) on Day 6.

### Real-time polymerase chain reaction (RT-PCR)

Briefly, a previously described protocol was used^52^. According to the manufacturer's directions, complementary deoxyribonucleic acid (cDNA) was synthesised by reverse transcriptase–polymerase chain reaction (RT-PCR) by using QuantiTect Reverse Transcription Kits (Qiagen, 205311) from RNA extracted from fHObs cultured on plasma treated and non-plasma treated hydrogels at day 8 of cell culture; RNeasy Protect Mini Kit (Qiagen, 74124). Expression of osteoblast genes was quantified using the QuantiFast SYBR Green PCR Kit (Qiagen, 204056) with Glyceraldehyde 3-phosphate dehydrogenase (GADPH), human alkaline phosphates (ALP), collagen type 1α1 (COL1A1), osteocalcin (OCN) and runt-related transcription factor 2 (RUNX2) primers, with GADPH serving as an internal control.The following amplification protocol was used: activation for 5 min at 95 °C, 40 cycles at 95 °C for 10 s, then 60 °C for 30 min. Each group analysed contained 3 samples. The 2−^ΔΔCT^ method was used to derive relative gene expression^53^. The results have been presented as fold change in gene expression. The biological control group consisted of fHObs cultured on an uncoated tissue culture plate (industry standard) and harvested a day after splitting to establish a baseline for gene expressions at the start of the experiment.

### Statistical analysis

Sample normality was tested using the Shapiro–Wilk test for continous variables where applicable and all tested populations followed a normal distribution. Differences between treated vs. non-treated hydrogels and fHObs vs. hESCs samples were compared using the two-tailed, independent *t* test by MedCalc version 19.2.1 (MedCalc Software bv, https://www.medcalc.org). The results are expressed as mean ± standard deviation except where otherwise stated. Results presented are from three or more independent experiments with three or more sample replicates. The threshold for statistical significance was set at a value of *p* < 0.05.

## Supplementary information

Supplementary Tables

## References

[1] Lee ES, Youn YS (2016). Albumin-based potential drugs: focus on half-life extension and nanoparticle preparation. J. Pharmaceut. Investig..

[2] Kratz F (2008). Albumin as a drug carrier: design of prodrugs, drug conjugates and nanoparticles. J Control Release.

[3] Elsadek B, Kratz F (2012). Impact of albumin on drug delivery–new applications on the horizon. J Control Release.

[4] Ong J, Zhao J, Justin AW, Markaki AE (2019). Albumin-based hydrogels for regenerative engineering and cell transplantation. Biotechnol Bioeng.

[5] Katarivas Levy G, Ong J, Birch MA, Justin AW, Markaki AE (2019). Albumin-enriched fibrin hydrogel embedded in active ferromagnetic networks improves osteoblast differentiation and vascular self-organisation. Polymers (Basel).

[6] Baler K, Michael R, Szleifer I, Ameer GA (2014). Albumin hydrogels formed by electrostatically triggered self-assembly and their drug delivery capability. Biomacromol.

[7] He XM, Carter DC (1992). Atomic structure and chemistry of human serum albumin. Nature.

[8] Carter DC, Ho JX (1994). Structure of serum albumin. Adv. Protein. Chem.

[9] Arabi SH (2018). Serum albumin hydrogels in broad pH and temperature ranges: characterization of their self-assembled structures and nanoscopic and macroscopic properties. Biomater. Sci..

[10] Chen J (2019). Preparation, characterization and application of a protein hydrogel with rapid self-healing and unique autofluoresent multi-functionalities. J. Biomed. Mater. Res. A.

[11] Zhang P, Sun F, Liu S, Jiang S (2016). Anti-PEG antibodies in the clinic: current issues and beyond PEGylation. J Control Release.

[12] Murata M, Tani F, Higasa T, Kitabatake N, Doi E (1993). Heat-induced transparent gel formation of bovine serum albumin. Biosci. Biotechnol. Biochem..

[13] Desmet T (2009). Nonthermal plasma technology as a versatile strategy for polymeric biomaterials surface modification: a review. Biomacromol.

[14] Li Y, Kim JH, Choi EH, Han I (2019). Promotion of osteogenic differentiation by non-thermal biocompatible plasma treated chitosan scaffold. Sci. Rep..

[15] Choi Y-R (2013). Surface modification of biphasic calcium phosphate scaffolds by non-thermal atmospheric pressure nitrogen and air plasma treatment for improving osteoblast attachment and proliferation. Thin Solid Films.

[16] Hsu SH, Lin CH, Tseng CS (2012). Air plasma treated chitosan fibers-stacked scaffolds. Biofabrication.

[17] Ko YM, Choi DY, Jung SC, Kim BH (2015). Characteristics of plasma treated electrospun polycaprolactone (PCL) nanofiber scaffold for bone tissue engineering. J. Nanosci. Nanotechnol..

[18] Moriguchi Y (2018). Impact of non-thermal plasma surface modification on porous calcium hydroxyapatite ceramics for bone regeneration. PLoS ONE.

[19] Zhang Q (2019). Air-plasma treatment promotes bone-like nano-hydroxylapatite formation on protein films for enhanced in vivo osteogenesis. Biomater Sci.

[20] Lee CM, Yang SW, Jung SC, Kim BH (2017). Oxygen plasma treatment on 3D-printed chitosan/gelatin/hydroxyapatite scaffolds for bone tissue engineering. J. Nanosci. Nanotechnol..

[21] Kinner B, Spector M (2002). Expression of smooth muscle actin in osteoblasts in human bone. J. Orthop. Res..

[22] Boraas LC, Guidry JB, Pineda ET, Ahsan T (2016). Cytoskeletal expression and remodeling in pluripotent stem cells. PLoS ONE.

[23] Woodruff MA, Jones P, Farrar D, Grant DM, Scotchford CA (2007). Human osteoblast cell spreading and vinculin expression upon biomaterial surfaces. J. Mol. Histol..

[24] Van Tam JK (2012). Mesenchymal stem cell adhesion but not plasticity is affected by high substrate stiffness. Sci. Technol. Adv. Mater..

[25] Kocgozlu L (2010). Selective and uncoupled role of substrate elasticity in the regulation of replication and transcription in epithelial cells. J. Cell. Sci..

[26] Gallego L, Junquera L, Meana A, Garcia E, Garcia V (2010). Three-dimensional culture of mandibular human osteoblasts on a novel albumin scaffold: growth, proliferation, and differentiation potential in vitro. Int. J. Oral Maxillofac. Implants.

[27] Ma X (2016). A biocompatible and biodegradable protein hydrogel with green and red autofluorescence: preparation, characterization and in vivo biodegradation tracking and modeling. Sci. Rep..

[28] Borzova VA (2016). Kinetics of thermal denaturation and aggregation of bovine serum albumin. PLoS ONE.

[29] Molodenskiy D (2017). Thermally induced conformational changes and protein-protein interactions of bovine serum albumin in aqueous solution under different pH and ionic strengths as revealed by SAXS measurements. Phys. Chem. Chem. Phys..

[30] Barone G (1995). Thermal denaturation of bovine serum albumin and its oligomers and derivativespH dependence. J. Therm. Anal..

[31] Matsudomi N, Rector D, Kinsella JE (1991). Gelation of bovine serum albumin and β-lactoglobulin; effects of pH, salts and thiol reagents. Food Chem..

[32] Shirahama H (2016). Fabrication of inverted colloidal crystal poly(ethylene glycol) scaffold: a three-dimensional cell culture platform for liver tissue engineering. J. Vis. Exp..

[33] Ng SS (2018). Human iPS derived progenitors bioengineered into liver organoids using an inverted colloidal crystal poly (ethylene glycol) scaffold. Biomaterials.

[34] Wang Y (2016). ECM proteins in a microporous scaffold influence hepatocyte morphology, function, and gene expression. Sci Rep.

[35] Fung J (2013). Defining normal liver stiffness range in a normal healthy Chinese population without liver disease. PLoS ONE.

[36] Colombo S (2011). Normal liver stiffness and its determinants in healthy blood donors. Dig Liver Dis.

[37] Barr RG (2015). Elastography assessment of liver fibrosis: society of radiologists in ultrasound consensus conference statement. Radiology.

[38] Guimarães CF, Gasperini L, Marques AP, Reis RL (2020). The stiffness of living tissues and its implications for tissue engineering. Nat. Rev. Mater..

[39] Amdursky N (2018). Elastic serum-albumin based hydrogels: mechanism of formation and application in cardiac tissue engineering. J. Mater. Chem. B.

[40] Fleischer S (2014). Albumin fiber scaffolds for engineering functional cardiac tissues. Biotechnol. Bioeng..

[41] Humphrey EJ (2017). Abstract 342: Serum albumin hydrogels alter excitation-contraction coupling in neonatal rat myocytes and human induced pluripotent stem cell derived cardiomyocytes. Circ. Res..

[42] Uehara N (2017). Osteoblast-derived Laminin-332 is a novel negative regulator of osteoclastogenesis in bone microenvironments. Lab. Invest..

[43] Jiang Z (2017). Laminin-521 promotes rat bone marrow mesenchymal stem cell sheet formation on light-induced cell sheet technology. Biomed Res Int.

[44] Mittag F (2012). Laminin-5 and type I collagen promote adhesion and osteogenic differentiation of animal serum-free expanded human mesenchymal stromal cells. Orthop Rev.

[45] van Leeuwen, J. P. T. M., van der Eerden, B. C. J., van de Peppel, J., Stein, G. S. & Lian, J. B. in *Osteoporosis* 161–207 (2013).

[46] Komori T (2017). Roles of Runx2 in skeletal development. Adv. Exp. Med. Biol..

[47] Czekanska EM, Stoddart MJ, Richards RG, Hayes JS (2012). In search of an osteoblast cell model for in vitro research. Eur. Cells Mater..

[48] Rutkovskiy A, Stenslokken KO, Vaage IJ (2016). Osteoblast differentiation at a glance. Med. Sci. Monit. Basic Res..

[49] Stein GS (2004). Runx2 control of organization, assembly and activity of the regulatory machinery for skeletal gene expression. Oncogene.

[50] Komori T (2008). Regulation of bone development and maintenance by Runx2. Front. Biosci..

[51] Varley MC (2016). Cell structure, stiffness and permeability of freeze-dried collagen scaffolds in dry and hydrated states. Acta Biomater.

[52] Katarivas Levy G, Birch MA, Brooks RA, Neelakantan S, Markaki AE (2019). Stimulation of human osteoblast differentiation in magneto-mechanically actuated ferromagnetic fiber networks. J. Clin. Med..

[53] Livak KJ, Schmittgen TD (2001). Analysis of relative gene expression data using real-time quantitative PCR and the 2(-Delta Delta C(T)) Method. Methods.

