# Supplementary material for: Functionalisation of a heat-derived and bio-inert albumin hydrogel with extracellular matrix by air plasma treatment
Source: Sci Rep. 2020 Jul 24;10:12429. doi: 10.1038/s41598-020-69301-7 (PMC7382478; doi:10.1038/s41598-020-69301-7)
Supplement: Supplementary file 1 — Supplementary Tables [file 41598_2020_69301_MOESM1_ESM.docx]

**FUNCTIONALISATION OF A HEAT-DERIVED AND BIO-INERT ALBUMIN HYDROGEL WITH EXTRACELLULAR MATRIX BY AIR PLASMA TREATMENT**

John Ong^1,2,3^, Junzhe Zhao^1,4^, Galit Katarivas Levy^1^, James Macdonald^1^, Alexander W. Justin^1^, Athina E. Markaki^1,*^

^1^Department of Engineering, University of Cambridge, Trumpington Street, Cambridge CB2 1PZ, UK

^2^East of England Gastroenterology Speciality Training Program, Fulbourn, Cambridge CB21 5XB, UK

^3^Department of Medicine, National University of Singapore, Kent Ridge Road, Singapore 119228

^4^Duke-NUS Medical School, 8 College Road, Singapore 169857

**Corresponding Authors:** Dr John Ong (jo401@ cam.ac.uk)

Dr Athina Markaki (am253@cam.ac.uk)

| **Albumin concentration (w/v)** | **Heating duration at 73-75^o^C** | | | | |
| --- | --- | --- | --- | --- | --- |
|  | **30min** | **1hr** | **2hr** | **4hr** | **Overnight** |
| 5.0% | HPB  G: Did not gel  C: N/A  HDF  G: Did not gel  C: N/A  HSA-DI  G: Did not gel  C: N/A | HPB  G: Did not gel  C: N/A  HDF  G: Did not gel  C: N/A  HSA-DI  G: Did not gel  C: N/A | HPB  G: Did not gel  C: N/A  HDF  G: Did not gel  C: N/A  HSA-DI  G: Did not gel  C: N/A | HPB  G: Did not gel  C: N/A  HDF  G: Did not gel  C: N/A  HSA-DI  G: Did not gel  C: N/A | HPB  G: Did not gel  C: N/A  HDF  G: Did not gel  C: N/A  HSA-DI  G: Did not gel  C: N/A |
| 7.5% | HPB  G: Did not gel  C: N/A  HDF  G: Did not gel  C: N/A  HDI  G: Did not gel  C: N/A | HPB  G: Did not gel  C: N/A  HDF  G: Did not gel  C: N/A  HDI  G: Did not gel  C: N/A | HPB  G: Gel in transition  C: Opaque  HDF  G: Gel in transition  C: Clear  HDI  G: Gel in transition  C: Clear | HPB  G: Gelled  C: Opaque  HDF  G: Gel in transition  C: Clear  HDI  G: Gel in transition  C: Clear | HPB  G: Gelled  C: Opaque  HDF  G: Gel in transition  C: Clear  HDI  G: Gel in transition  C: Clear |
| 10.0% | HPB  G: Gel in transition  C: Opaque  HDF  G: Gel in transition  C: Translucent  HDI  G: Gel in transition  C: Clear | HPB  G: Gelled  C: Opaque  HDF  G: Gel in transition  C: Translucent  HDI  G: Gel in transition  C: Clear | HPB  G: Gelled  C: Opaque  HDF  G: Gelled  C: Translucent  HDI  G: Gelled  C: Clear | HPB  G: Gelled  C: Opaque  HDF  G: Gelled  C: Translucent  HDI  G: Gelled  C: Clear | HPB  G: Gelled  C: Opaque  HDF  G: Gelled  C: Translucent  HDI  G: Gelled  C: Clear |
| 12.5% | HPB  G: Gelled  C: Opaque  HDF  G: Gel in transition  C: Translucent  HDI  G: Gel in transition  C: Translucent | HPB  G: Gelled  C: Opaque  HDF  G: Gelled  C: Nearly opaque  HDI  G: Gel in transition  C: Translucent | HPB  G: Gelled  C: Opaque  HDF  G: Gelled  C: Nearly opaque  HDI  G: Gelled  C: Nearly opaque | HPB  G: Gelled  C: Opaque  HDF  G: Gelled  C: Opaque  HDI  G: Gelled  C: Nearly opaque | HPB  G: Gelled  C: Opaque  HDF  G: Gelled  C: Opaque  HDI  G: Gelled  C: Opaque |
| 15.0% | HPB  G: Gelled  C: Opaque  HDF  G: Gel in transition  C: Nearly opaque  HDI  G: Gel in transition  C: Translucent | HPB  G: Gelled  C: Opaque  HDF  G: Gelled  C: Opaque  HDI  G: Gelled  C: Nearly opaque | HPB  G: Gelled  C: Opaque  HDF  G: Gelled  C: Opaque  HDI  G: Gelled  C: Nearly opaque | HPB  G: Gelled  C: Opaque  HDF  G: Gelled  C: Opaque  HDI  G: Gelled  C: Opaque | HPB  G: Gelled  C: Opaque  HDF  G: Gelled  C: Opaque  HDI  G: Gelled  C: Opaque |
| 20.0% | HPB  G: Gelled  C: Opaque  HDF  G: Gelled  C: Nearly opaque  HDI  G: Gel in transition  C: Nearly opaque | HPB  G: Gelled  C: Opaque  HDF  G: Gelled  C: Opaque  HDI  G: Gelled  C: Opaque | HPB  G: Gelled  C: Opaque  HDF  G: Gelled  C: Opaque  HDI  G: Gelled  C: Opaque | HPB  G: Gelled  C: Opaque  HDF  G: Gelled  C: Opaque  HDI  G: Gelled  C: Opaque | HPB  G: Gelled  C: Opaque  HDF  G: Gelled  C: Opaque  HDI  G: Gelled  C: Opaque |

**Supplementary Table S1**. The effect of albumin concentration (w/v) on hydrogel formation (G) and clarity (C). HPB = human albumin dissolved in phosphate buffered saline, HDF = human albumin dissolved in DMEM/F12 and HDI = human albumin dissolved in de-ionized water. Heating temperature was maintained between. Gel in transition describes hydrogels in which the critical point had been reached but the phase transition was incomplete.

| **Temperature** | **Heating duration** | | | | |
| --- | --- | --- | --- | --- | --- |
|  | 0.5hr | 1hr | 2hr | 4hr | Overnight |
| 40^o^C | HPB: Did not gel  HDF: Did not gel  HDI: Did not gel | HPB: Did not gel  HDF: Did not gel  HDI: Did not gel | HPB: Did not gel  HDF: Did not gel  HDI: Did not gel | HPB: Did not gel  HDF: Did not gel  HDI: Did not gel | HPB: Did not gel  HDF: Did not gel  HDI: Did not gel |
| 45^o^C | HPB: Did not gel  HDF: Did not gel  HDI: Did not gel | HPB: Did not gel  HDF: Did not gel  HDI: Did not gel | HPB: Did not gel  HDF: Did not gel  HDI: Did not gel | HPB: Did not gel  HDF: Did not gel  HDI: Did not gel | HPB: GT (O)  HDF: GT (O)  HDI: Did not gel |
| 50^o^C | HPB: Did not gel  HDF: Did not gel  HDI: Did not gel | HPB: Did not gel  HDF: Did not gel  HDI: Did not gel | HPB: GT (T)  HDF: GT (T)  HDI: Did not gel | HPB: GT (T)  HDF: GT (T)  HDI: Did not gel | HPB: GT (O)  HDF: GT (O)  HDI: Did not gel |
| 55^o^C | HPB: Did not gel  HDF: Did not gel  HDI: Did not gel | HPB: Did not gel  HDF: Did not gel  HDI: Did not gel | HPB: GT (T)  HDF: GT (T)  HDI: Did not gel | HPB: GT (O)  HDF: GT (T)  HDI: Did not gel | HPB: Gelled (O)  HDF: Gelled (O)  HDI: Did not gel |
| 60^o^C | HPB: Did not gel  HDF: Did not gel  HDI: Did not gel | HPB: Did not gel  HDF: Did not gel  HDI: Did not gel | HPB: GT (T)  HDF: GT (T)  HDI: Did not gel | HPB: GT (O)  HDF: GT (T)  HDI: GT (C) | HPB: Gelled (O)  HDF: Gelled (O)  HDI: GT (C) |
| 65^o^C | HPB: Did not gel  HDF: Did not gel  HDI: Did not gel | HPB: Did not gel  HDF: Did not gel  HDI: Did not gel | HPB: Gelled (O)  HDF: GT (T)  HDI: GT (C) | HPB: Gelled (O)  HDF: Gelled (T)  HDI: GT (C) | HPB: Gelled (O)  HDF: Gelled (O)  HDI: GT (C) |
| 70^o^C | HPB: GT (O)  HDF: GT (T)  HDI: GT (C) | HPB: GT (O)  HDF: GT (T)  HDI: GT (C) | HPB: Gelled (O)  HDF: Gelled (T)  HDI: Gelled (C) | HPB: Gelled (O)  HDF: Gelled (T)  HDI: Gelled (C) | HPB: Gelled (O)  HDF: Gelled (O)  HDI: Gelled (C) |
| 75^o^C | HPB: GT (O)  HDF: GT (T)  HDI: GT (C) | HPB: GT (O)  HDF: GT (T)  HDI: GT (C) | HPB: Gelled (O)  HDF: Gelled (T)  HDI: Gelled (C) | HPB: Gelled (O)  HDF: Gelled (T)  HDI: Gelled (C) | HPB: Gelled (O)  HDF: Gelled (O)  HDI: Gelled (C) |
| 80^o^C | HPB: Gelled (O)  HDF: Gelled (O)  HDI: Gelled (T) | HPB: Gelled (O)  HDF: Gelled (O)  HDI: Gelled (T) | HPB: Gelled (O)  HDF: Gelled (O)  HDI: Gelled (T) | HPB: Gelled (O)  HDF: Gelled (O)  HDI: Gelled (T) | HPB: Gelled (O)  HDF: Gelled (O)  HDI: Gelled (T) |

**Supplementary Table S2**. The effect of temperature and heating duration on hydrogel formation. HPB = 10% w/v Human serum albumin dissolved in phosphate buffered saline, HDF = 10% w/v human serum albumin dissolved in DMEM/F12, and HDI = 10% w/v human serum albumin dissolved in de-ionized water. Additional sodium chloride was not added to the solvents. Hydrogel clarity = Clear (C), Translucent (T) and Opaque (O). GT = describes hydrogels in which the critical point had been reached but the phase transition was incomplete. These gels frequently were unstable and easily damaged on any physical contact.

| **Heating duration**  **(73 ^o^C - 75^o^C)** | **Increase in NaCl concentration** | | | | | |
| --- | --- | --- | --- | --- | --- | --- |
|  | **0mM** | **30mM** | **60mM** | **90mM** | **120mM** | **150mM** |
| 0.5 hour | HPB  C: Opaque  M: *  S: Unstable  HDF  C: Translucent  M: Clear  S: Unstable  HDI  C: Clear  M: Clear  S: Unstable | HPB  C: Opaque  M: *  S: Unstable  HDF  C: Translucent  M: Clear  S: Unstable  HDI  C: Clear  M: Macroporous  S: Unstable | HPB  C: Opaque  M: *  S: Unstable  HDF  C: Near opaque  M: Clear  S: Unstable  HDI  C: Clear  M: Clear  S: Unstable | HPB  C: Opaque  M: *  S: Unstable  HDF  C: Opaque  M: *  S: Stable  HDI  C: Clear  M: Clear  S: Unstable | HPB  C: Opaque  M: *  S: Unstable  HDF  C: Opaque  M: *  S: Stable  HDI  C: Clear  M: Clear  S: Unstable | HPB  C: Opaque  M: *  S: Unstable  HDF  C: Opaque  M: *  S: Stable  HDI  C: Clear  M: Clear  S: Unstable |
| 1 hour | HDF  C: Translucent  M: Clear  S: Unstable  HDI  C: Clear  M: Clear  S: Unstable | HDF  C: Translucent  M: Clear  S: Unstable  HDI  C: Clear  M: Macroporous  S: Unstable | HDF  C: Near opaque  M: Clear  S: Stable  HDI  C: Clear  M: Clear  S: Unstable | HDF  C: Opaque  M: *  S: Stable  HDI  C: Translucent  M: Macroporous  S: Unstable | HDI  C: Translucent  M: Macroporous  S: Unstable | HDI  C: Translucent  M: Macroporous  S: Unstable |
| 2 hours | HDF  C: Translucent  M: Clear  S: Unstable  HDI  C: Clear  M: Clear  S: Unstable | HDF  C: Translucent  M: Clear  S: Stable  HDI  C: Translucent  M: Macroporous  S: Unstable | HDF  C: Near opaque  M: Clear  S: Stable  HDI  C: Translucent  M: Macroporous  S: Unstable | HDF  C: Opaque  M: *  S: Stable  HDI  C: Translucent  M: Macroporous  S: Unstable | HDI  C: Translucent  M: Macroporous  S: Unstable | HDI  C: Translucent  M: Macroporous  S: Unstable |
| 4 hours | HDF  C: Translucent  M: Clear  S: Unstable  HDI  C: Clear  M: Clear  S: Unstable | HDF  C: Translucent  M: Clear  S: Stable  HDI  C: Translucent  M: Macroporous  S: Unstable | HDF  C: Near opaque  M: Clear  S: Stable  HDI  C: Translucent  M: Clear  S: Unstable | HDF  C: Opaque  M: *  S: Stable  HDI  C: Translucent  M: Macroporous  S: Unstable | HDI  C: Translucent  M: Macroporous  S: Unstable | HDI  C: Translucent  M: Macroporous  S: Unstable |
| Overnight | HDF  C: Translucent  M: Clear  S: Unstable  HDI  C: Clear  M: Clear  S: Unstable | HDF  C: Near opaque  M: Clear  S: Stable  HDI  C: Translucent  M: Macroporous  S: Unstable | HDF  C: Opaque  M: Clear  S: Stable  HDI  C: Translucent  M: Clear  S: Unstable | HDF  C: Opaque  M: *  S: Stable  HDI  C: Translucent  M: Macroporous  S: Unstable | HDI  C: Translucent  M: Macroporous  S: Unstable | HDI  C: Translucent  M: Macroporous  S: Unstable |

**Supplementary Table S3**. The effect of sodium chloride (NaCl) on hydrogel clarity (C), microscopic appearance (M) and stability (S) of thermally derived human albumin hydrogels. HPB = 10% w/v human serum albumin in phosphate buffered saline, HDF = 10% human serum albumin w/v in DMEM/F12, and HDI = 10% w/v human serum albumin in de-ionized water. Unable to determine = *. Hydrogels that damaged easily during handling or air-plasma treatment were described as unstable. Experiments on HSA-PBS were discontinued because opaque hydrogels were obtained during the shortest duration of heating.

|  | **Contact angle of plasma treated surfaces (°)** | **Contact angle of untreated surfaces (°)** |
| --- | --- | --- |
| Measurement 1 | 48.05 | 65.35 |
| Measurement 2 | 36.83 | 65.66 |
| Measurement 3 | 45.32 | 67.94 |
| Measurement 4 | 48.05 | 67.45 |
| Measurement 5 | 50.77 | 65.34 |
| Measurement 6 | 38.51 | 69.04 |
| Measurement 7 | 50.77 | - |
| Measurement 8 | 47.2 | - |
| Mean ± SD | 45.7 ± 5.3 | 66.8 ± 1.6 |
| **Unpaired t-test (treated vs. untreated samples) = *p* < 0.0001** | | |

**Supplementary Table S4**. Contact angle measurements of plasma treated and untreated HSA-DMEM/12 hydrogel surfaces.

| **Air plasma treatment duration** | **0min** | **0.5min** | **1min** | **1.5min** | **2min** | **2.5min** | **3min** |
| --- | --- | --- | --- | --- | --- | --- | --- |
| High intensity  (29.6W) | CC: nil  BM: clear | CC: nil  BM: clear | CC: 5-10%  BM: clear | CC: 10-15%  BM: clear | CC: 35-45%  BM: clear | CA: *  BM: mp | CA: *  BM: mp |
| Low Intensity  (10.2W) | CC: nil  BM: clear | CC: nil  BM: clear | CC: nil  BM: clear | CC: nil  BM: clear | CC: nil  BM: clear | CA: *  BM: mp | CA: *  BM: mp |

**Supplementary Table S5**. Effect of air-plasma treatment time and intensity on (i) cell attachment measured by cell confluence, and (ii) microscopic appearance of thermally derived human albumin hydrogels coated with growth factor reduced Matrigel (Geltrex). CC = Cell confluence 48 hours after cell seeding. Cell confluence in Geltrex-coated, polystyrene tissue culture plates (control) was 30-40% at 48 hours after cell seeding. BM = Appearance under brightfield microscopy: clear or macroporous (mp). Results are based on cells grown on plasma treated, surface-coated 10% HSA-DMEM/F12 hydrogels.
